# A rare case of fetal bilateral Wilms' tumor in horseshoe kidney: A case report

**DOI:** 10.1016/j.ijscr.2025.111619

**Published:** 2025-07-08

**Authors:** Abebe Melis, Teketel Tadesse, Samson Jemal, Worku Ketema

**Affiliations:** a Hawassa university college of medicine and health science, department of pathology, Hawassa, Ethiopia; b Naol Primary Hospital, Hawassa, Ethiopia; c Hawassa university college of medicine and health science, department of pediatrics and child health, Hawassa, Ethiopia

**Keywords:** Fetal bilateral Wilms' tumor, Horseshoe kidney, Case report

## Abstract

**Introduction and importance:**

Wilms' tumor is the most common malignant kidney tumor in children. The horseshoe kidney is the most common renal fusion malformation. However, Wilms' tumor is rarely identified in horseshoe kidney patients. The incidence of horseshoe kidney is about 1 in 400 cases. Wilms' tumor or Nephrogenic rests can occur in both kidneys, termed bilateral disease, found in only 5–8 % of cases. Management of bilateral Wilms' tumor presents a major clinical challenge in terms of maximizing survival, preserving renal function, and understanding underlying genetic risk.

**Case presentation:**

Our case is a 28-year-old primigravid female patient, who came for the complaint of decreased abdominal girth and decreased fetal moment. Obstetric ultrasound concluded severe oligohydramnios + renal mass and safe termination was done. Subsequent examinations of the autopsy revealed bilateral Wilms tumor in horseshoe kidney.

**Clinical discussion:**

Ultrasound is used to diagnose horseshoe kidneys, whereas Computed tomography and magnetic resonance imaging are often used for staging purposes. Histopathological analysis is the current gold standard for diagnosing Wilms' tumor. Surgery, chemotherapy, and radiotherapy are also used to treat Wilms' tumor.

**Conclusion:**

Fetal Wilms' tumor (WT) is extremely rare, but advances in fetal imaging have led to increased detection of such cases. Managing these cases remains challenging due to the complexity of treatment and the needed for preserving renal function. Prenatal ultrasound is an essential diagnostic tool for early detection and intervention.

## Highlights

• **Rare Case:** Fetal bilateral Wilms' tumor in a horseshoe kidney diagnosed in a 28-year-old primigravid patient from Sidama, Ethiopia, emphasizing challenges in managing congenital anomalies during pregnancy.
• **Clinical Features:** Decreased fetal movement, severe oligohydramnios, and abdominopelvic mass on prenatal ultrasound noted.
• **Diagnosis/Intervention:** Prenatal suspicion of nephroblastoma; medical termination due to severe congenital defects, with histopathologic confirmation.
• **Histopathology:** Autopsy revealed fused kidneys (horseshoe) with multicystic lesions and triphasic Wilms' tumor on microscopy.
• **Outcome:** Uncomplicated maternal recovery; discharged with follow-up guidance.
• **Significance:** Underscores prenatal imaging role in diagnosing rare renal tumors, particularly in resource-limited settings.

## 1 Introduction

Wilms' tumor (WT) also known as Nephroblastoma, is the most commonly detected pediatric malignant renal mass, accounting for 87 % of all malignant renal masses and 7 % of all malignant tumors found in children [1,2]. The tumor is associated with undifferentiated embryonic lesions called nephrogenic rests (NRs) or, when diffuse, nephroblastomatosis. WT or NRs can occur in both kidneys, termed bilateral disease, found in only 5–8 % of cases [3]. Bilateral disease can be synchronous (both kidneys affected at the same time) or metachronous (one affected after the other), which occurs in 6.3 and 0.85 % WT patients respectively [4].The median age at which this tumor is diagnosed is three years [5]. Prenatal detection of Wilms tumor is extremely rare. Prenatal ultrasonography and fetal magnetic resonance imaging (MRI) has been found to be helpful in diagnosis of renal masses [6]. Wilms' tumor is linked to several syndromes, such as WAGR syndrome (Wilms' tumor, aniridia, genitourinary abnormalities, and spectrum of developmental delays), Beckwith-Wiedemann syndrome, Denys-Drash syndrome, and Edwards or Perlman syndrome [7]. Some cases are associated with prenatal polyhydramnios and fetal hydrops [8,9].Horseshoe kidney occurs in around 1 in 500 instances, with a higher incidence in males [10]. Nevertheless, the presence of Wilms' tumor in horseshoe kidney is very uncommon, with an estimated occurrence rate of roughly 0.4–0.9 % of all Wilms' tumors [11].

This case report is written following the SCARE criteria [12].

## 2 Case presentation

Our case is a 28-year-old primigravid female patient from the Sidama region, Ethiopia, who came for the complaint of decreased abdominal girth and decreased fetal moment. Other prenatal history was unremarkable.

On physical examination, her vital signs were all normal. Abdominal examination revealed 18-week sized gravid uterus.

On laboratory tests: Work-up for complete blood count, liver and renal function tests were within the normal range. Her blood group and Rh were AB negative.

Obstetric Ultrasound: Singleton intra-uterine pregnancy, alive with Biparietal diameter (BPD) measuring 22 + 3, Femoral length (FL) 22 + 6, Abdominal circumference (AC): 31 weeks. There is 8 × 7 cm measuring abdominopelvic mass, abdominal contents are pushed peripherally by the mass. Placenta fundal with no measurable liquor. Index: Severe oligohydramnios + with renal mass secondary to? Wilms tumor top on the list.

Hence, safe termination was decided for the indication of incompatible congenital defects based on the existing trends. We put her on maintenance fluid with Normal saline and safe medical termination was done. Sample from fetus and products of conceptus were sent for histopathologic examination.

**Fig. 1:**
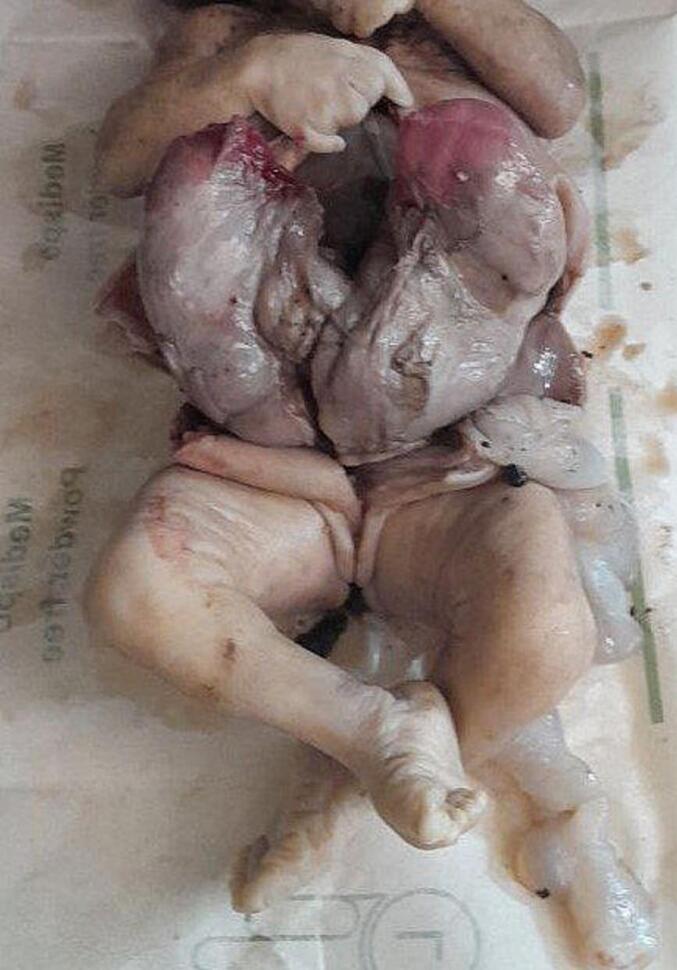
Gross evaluation: Bilateral enlarged kidneys with fused lower pole.

**Fig. 2:**
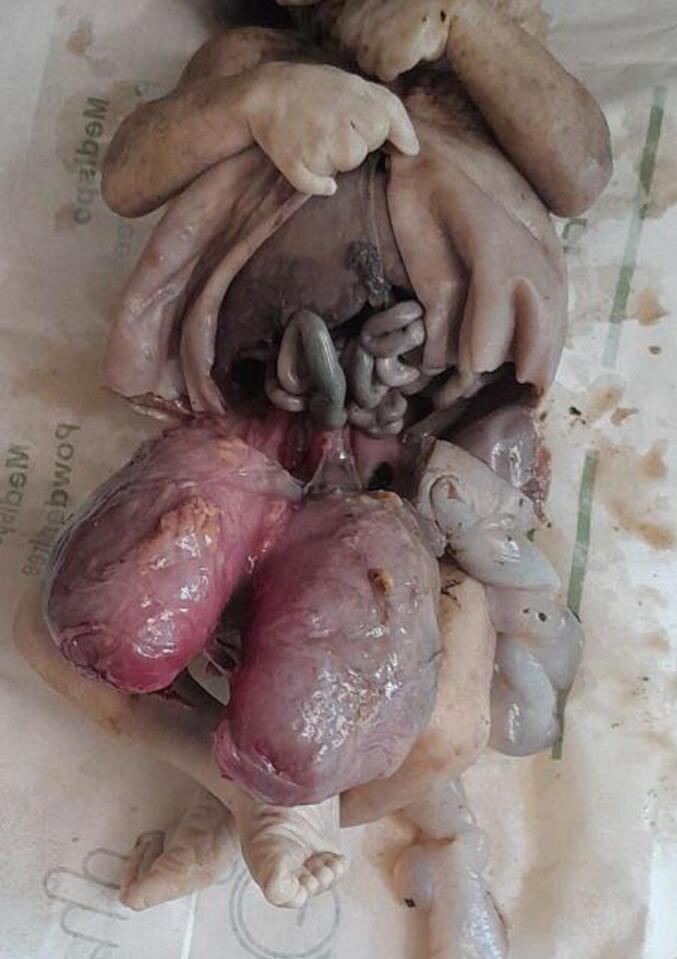
Gross evaluation: Bilateral enlarged kidneys with fused lower pole.

**Fig. 3:**
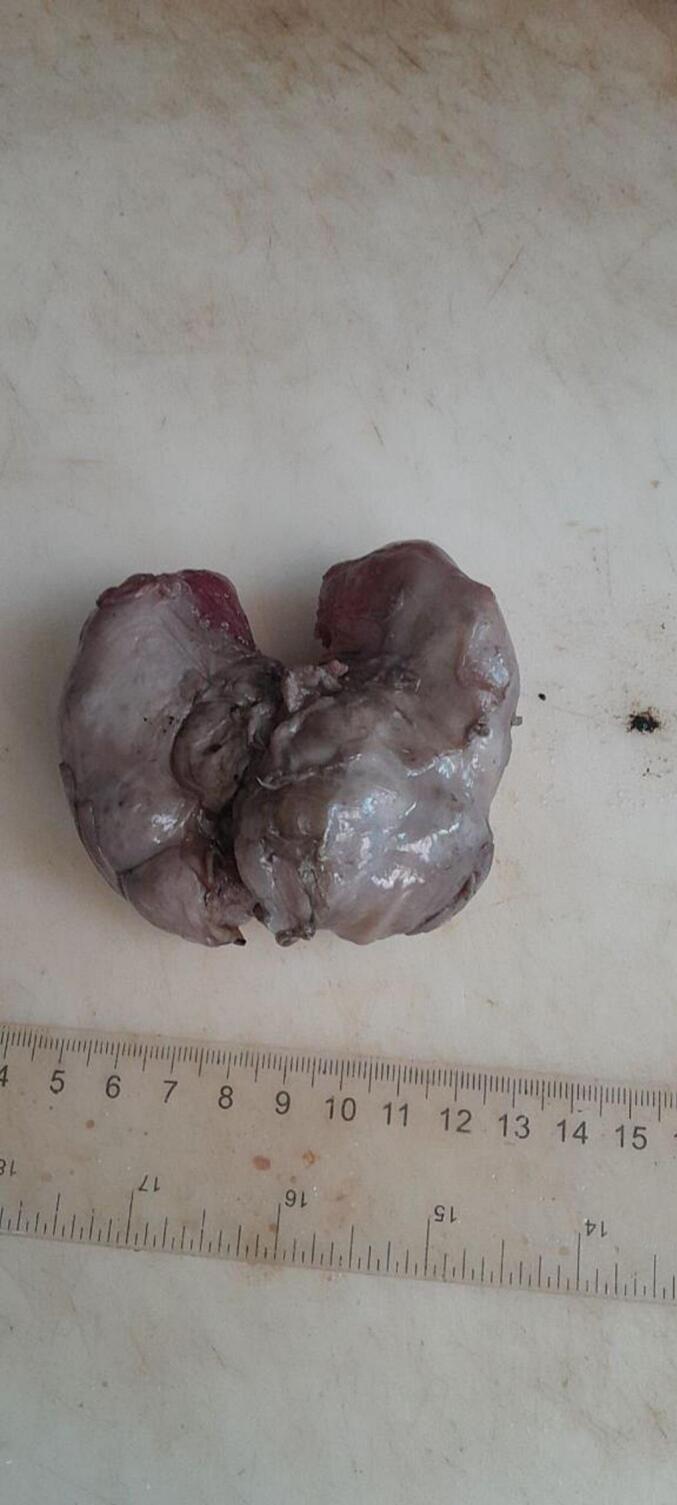
Gross evaluation: Bilateral enlarged kidneys with fused lower pole.

**Fig. 4:**
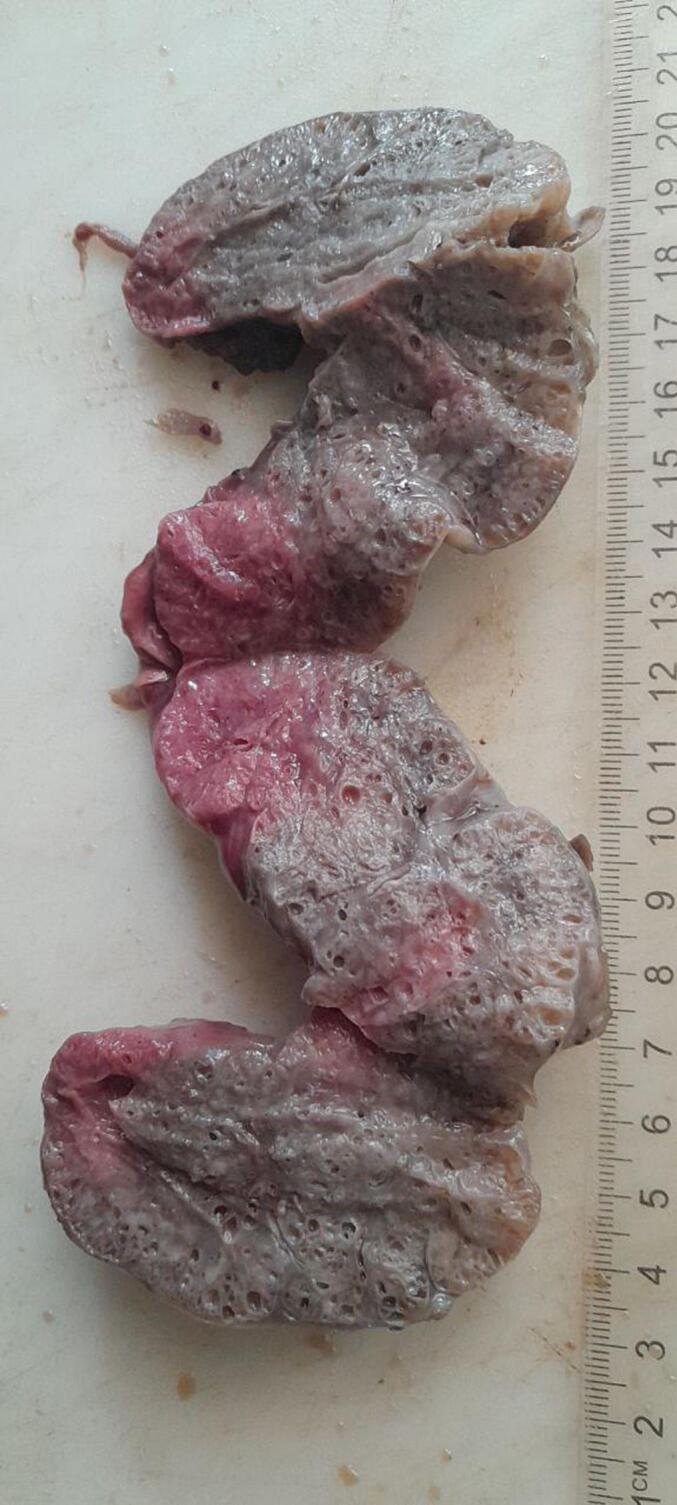
Gross evaluation: Cut surface revealing multicystic spaces effacing the renal parenchyma.

Subsequent examinations of the autopsy revealed a bilaterally enlarged kidneys with fused lower pole with cut surface revealing multicystic spaces effacing the renal parenchyma (Fig. 1, Fig. 2, Fig. 3, Fig. 4).

**Fig. 5:**
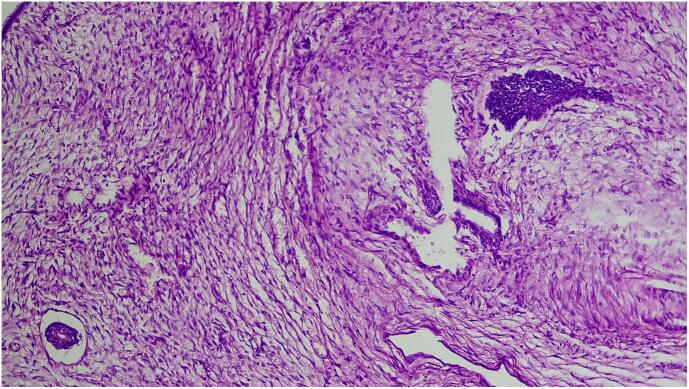
Microscopic examination (H&E stain). Medium power microscopic examination demonstrating small abortive tubules and focal blastemal element.

**Fig. 6:**
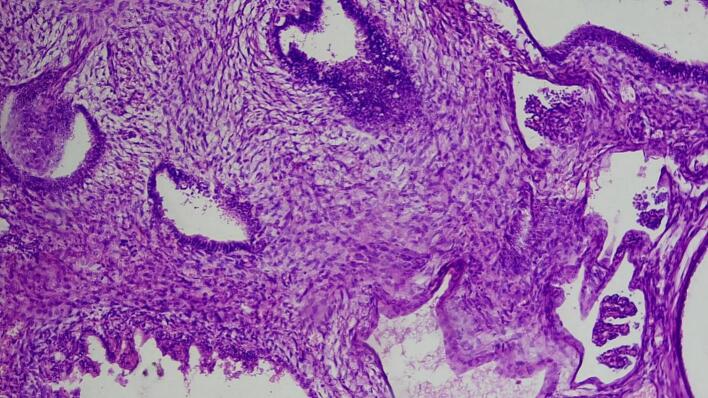
Microscopic examination (H&E stain). Medium power microscopic examination revealing variable sized tubules set in hypo and hypercellular stromal component.

**Fig. 7:**
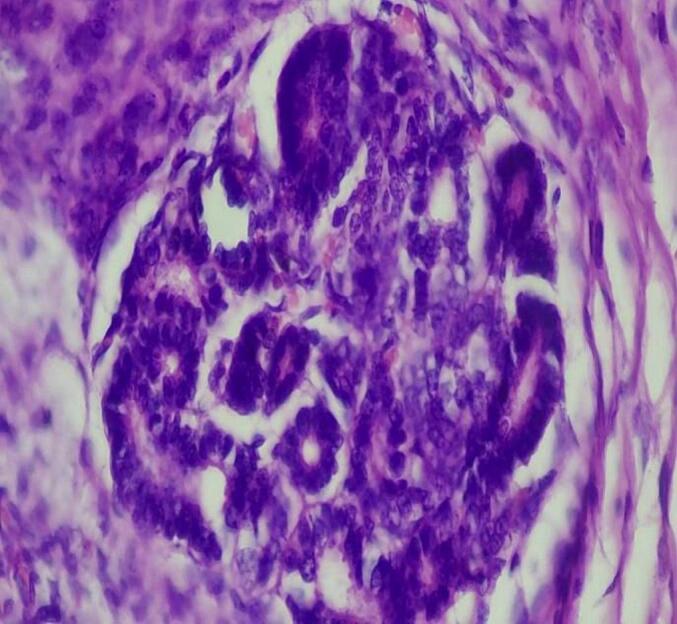
Microscopic examination (H&E stain). Higher power view showing epithelial elements.

**Fig. 8:**
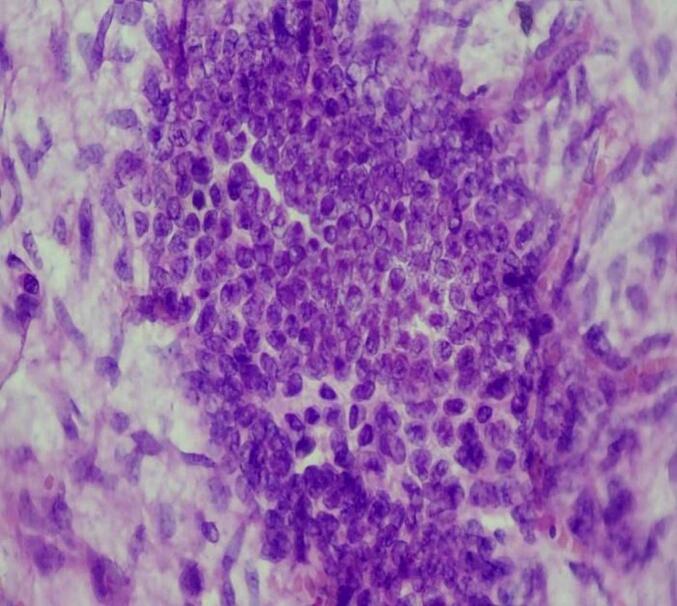
Microscopic examination (H&E stain). High power view showing blastemal elements.

Microscopic examination shows triphasic proliferation of blastemal, epithelial and stromal components which is consistent with Wilm's tumor (Fig. 5, Fig. 6, Fig. 7, Fig. 8).

The mother had a smooth post-treatment course and was discharged on the 2nd day with oral antibiotics and advice for subsequent follow-ups and pregnancy.

## 3 Discussion

During the fetal period, the kidneys develop and ascend, developing first in the pelvis and then gradually ascending into position below the thorax, on either side of the lumbar spine. During this ascending process, the kidneys also rotate, which typically occurs by the gestational ninth week [13]. Renal fusion anomalies may occur during this process [14]. The isthmus of the horseshoe kidney may contain functioning renal parenchyma or a fibrous band [15]. In up to 80 % of cases of horseshoe kidney, the isthmus contains functional renal parenchyma tissue, and in >90 % of cases, fusion occurs at the lower pole [13]. Patients with horseshoe kidney are often asymptomatic and are typically discovered incidentally, often due to symptoms secondary to pelvic ureteric junction obstruction and infection [16]. These patients are thought to be at increased risk of developing malignancies, such as renal cell carcinoma, Wilms' tumor, and carcinoids, among which renal cell carcinoma is the most common [14]. However, Wilms' tumor is the most common malignant kidney tumor identified in children [17]. The risk of Wilms' tumor in children with horseshoe kidney is 2–6 times that of children in the general population [18]. Approximately 50 % of Wilms' tumors in horseshoe kidney develop from the isthmus, likely due to the abnormal proliferation of metanephric blastema in the isthmus [14]. The same anomaly that causes the development of horseshoe kidney may also lead to the development of Wilms' tumor [19]. Patients with Wilms' tumors are often asymptomatic; approximately 10 % are discovered incidentally after trauma, whereas 25 % present with microscopic hematuria or hypertension secondary to renin production [20]. Ultrasound is used to diagnose horseshoe kidney, whereas Computed tomography (CT) and magnetic resonance imaging (MRI) are often used for staging purposes [21]. On ultrasound, the mass presents as a large renal mass, which can be either solid or cystic, with large hypoechoic areas due to central necrosis and cyst formation. Areas characterized by fat deposits, calcification, or hemorrhage may appear [20]. On CT, the tumors are lower density and enhance less than the normal renal parenchyma. Tumors are often characterized by heterogeneous contrast enhancement and may feature punctuated calcifications [22]. On MRI, the tumors have low signal intensity on T1-weighted images, with either low or high signal intensity on T2-weighted images and restricted diffusivity on diffusion-weighted images. CT is also used for the detection of lung metastasis or local recurrence [20]. Wilms' tumors contain variable quantities of embryonic renal elements, such as blastema, epithelium, and stroma [23]. Wilms' tumor can be divided into 2 types, based on prognosis: favorable (over 90 %) and unfavorable (6–10 %) [20]. Histopathological analysis is the current gold standard for diagnosing Wilms' tumor. Germline aberration of *WT1* is clearly associated with increase in bilateral disease as the overall rate of bilateral WT is 5 % whereas patients with DDS show incidence of 20 %, and WAGR 17 % but due absence of the test it was not done in this patient. Surgery, chemotherapy, and radiotherapy are also used to treat Wilms' tumor [17]. The National Wilms Tumor Study Group (NWTSG)/Children's Oncology Group (COG) and the International Society of Pediatric Oncology (SIOP) have established the major guidelines regarding the management of Wilms' tumor [24]. SIOP recommends using preoperative chemotherapy to reduce the tumor size and prevent intraoperative spillage due to tumor rupture [17]. In contrast, the NWTSG/COG recommends the application of primary surgery before any adjuvant treatments [24]. The overall survival of children with Wilms' tumor in horseshoe kidney appears to be similar to that among children with Wilms' tumor in normal kidneys [11].

## 4 Conclusion

Fetal Wilms' tumor (WT) is extremely rare, but advances in fetal imaging have led to increased detection of such cases. Managing these cases remains challenging due to the complexity of treatment and the needed for preserving renal function. Prenatal ultrasound is an essential diagnostic tool for early detection and intervention.

## Informed consent

Written informed consent was obtained from the patient, by their native language, for publication of non-identifying information including accompanying intraoperative images. A copy of the written consent is available for review by the Editor-in-Chief of this journal on request.

## Ethical approval

Ethical approval is deemed unnecessary by the Hawassa university college of medical and health science ethical committee as this is a single case encountered during practice and it doesn’t involve human or animal experiment.

## Funding

No funding was provided for this case report.

## Author contribution

Abebe Melis Nisro, MD - Study concept and design, writing the paper, literature review and editing and critical review of the paper.

Teketel Tadesse Geremew, MD - Study concept and design, writing the paper, literature review and editing and critical review of the paper.

Samson Jemal and Worku Ketema, MD - Involved in acquisition of data, literature review of the paper, writing and drafting the paper, editing and critical review of the paper.

## Guarantor

Abebe Melis Nisro, MD.

## Research registration number

1.Name of the registry: Open Science Framework (OSF) Registries 2

2.Unique identifying number or registration ID: 10.17605/OSF.IO/AHUXP 3

3.Hyperlink to your specific registration: https://doi.org/10.17605/OSF.IO/AHUXP

## Conflict of interest statement

No conflict of interest between the authors.

## Data Availability

The data used to support the findings of this case report are available from the corresponding author upon reasonable request.
